# B cells defined by immunoglobulin isotypes

**DOI:** 10.1093/cei/uxac091

**Published:** 2022-10-05

**Authors:** Louisa Katherine James

**Affiliations:** Blizard Institute, Queen Mary University of London, London E1 2AT, UK

**Keywords:** antibodies, B cells, immunoglobulins, Fc receptors, memory

## Abstract

The ability of B cells to generate antibodies and provide long-lived protective immunity is the cornerstone of vaccination and has contributed to the success of modern medicine. The nine different antibody subclasses produced by humans have effector functions that differ according to antigen type and route of exposure. Expression of the appropriate isotype is critical for effective humoral immunity, and it is becoming clear that subclass specificity is to some extent reflected at the cellular level. Understanding the mechanisms that govern the induction, expansion, and maintenance of B cells expressing different antibody subclasses informs the strategic manipulation of responses to benefit human health. This article provides an overview of the mechanisms by which the different human antibody subclasses regulate immunity, presents an update on how antibody subclass expression is regulated at the cellular level and highlights key areas for future research.

## Teaser

Expression of the appropriate antibody isotype is critical for effective humoral immunity. Subclass specificity is to some extent reflected at the cellular level and better understanding of the mechanisms that govern the induction, expansion, and maintenance of B cells expressing different antibody subclasses could be valuable to for developing B cell targeting treatments.

## Introduction

The incredible diversity of antibody responses allows our immune system to react to an unlimited number of antigens with high specificity and is central to protective immunity. As well as protecting us from pathogens, antibodies maintain homeostasis by regulating commensal organisms at mucosal surfaces and facilitating the clearance of apoptotic cells. All of this is achieved through a broad range of effector functions that can take place throughout the human body. In addition to neutralization, which is entirely dependent on the variable region, these effector functions depend on the Fc portion of antibodies interacting with cell surface receptors. Localized patterns of subclass expression and receptor distribution regulate antibody responses appropriately for site-specific threats. This specialization is most evident in selective antibody deficiencies, where the absence of an individual subclass can increase susceptibility to specific pathogens. Similarly, dysregulation of subclass expression can promote disease, as is the case in allergies and various autoimmune pathologies. The selection of antibody class is strongly influenced by the site of antigen exposure and the context in which B cell activation occurs. In turn, the selection of antibody class can modify B cell fate and function.

B cells are generated in the bone marrow continuously throughout life, emerging as antigen-naïve B cells that express membrane IgM as a B cell receptor. Alternative splicing allows the transcription of IgD with an identical specificity that is co-expressed alongside membrane IgM. Mature naïve IgM+IgD+ B cells circulate through the follicles of secondary lymphoid tissues until they encounter antigen. Multi-valent antigens and/or signaling from other co-receptors such as toll-like receptors can drive a T-cell independent response. Otherwise, B cells will migrate to the T:B border of the follicle where T-dependent responses are initiated. Activation of B cells results in a burst of proliferation during which class switch recombination (CSR) can occur and germinal centre responses are initiated. During germinal centre responses, B cells further diversify their antigen receptors through somatic hypermutation and are ultimately selected to enter the memory pool as either plasma cells or memory B cells. Plasma cells migrate to survival niches from where they can secrete antibody continuously for a lifetime [1]. Memory B cells appear to be equally long-lived [2, 3], and either recirculate through the periphery or take up residence in tissues where they can respond rapidly to secondary infection. The decision to differentiate into memory B cells or plasma cells is influenced by several factors, including the isotype of the antibody expressed. Furthermore, the function of memory B cells—and to some extent plasma cells—is influenced by the subclass of antibodies expressed. Memory B cells can undergo further diversification during secondary responses, including re-entry into germinal centres and subsequent class switching. Regulation of antibody subclass expression is therefore highly relevant to the outcome of both primary and secondary responses.

This review will highlight the key roles different antibody subclasses play in specific aspects of immunity, the mechanisms that regulate their distribution and production, as well as discussing the evidence for intrinsic cellular properties based on antibody isotype. The importance of regulating antibody production is highlighted by the clinical phenotypes of selective deficiencies, but also when responses are dysregulated such as in allergy and autoimmune conditions. Better understanding of how antibody responses are regulated at the cellular level will present new opportunities for targeting B cells associated with antibody-mediated disease.

## Diversity of antibody effector functions

**Figure 1:**
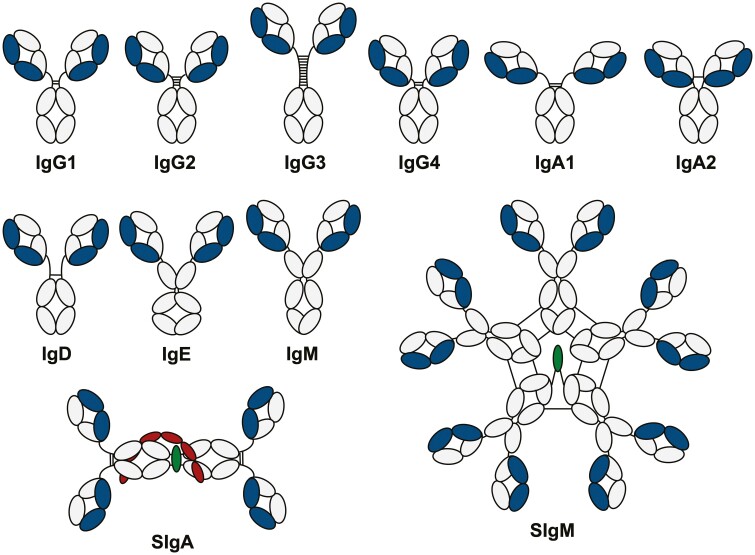
Domain structure of human immunoglobulins. Humans produce nine different antibody subclasses: IgG1-4, IgA1-2, IgD, IgE, and IgM. Each consists of two identical heavy chains (grey) which comprise an N-terminal V_H_ domain and three C_H_ domains with a hinge between C_H_1 and C_H_2 (or four C_H_ domains in the case of IgE and IgM, which lack hinges). The heavy chains are linked to two identical light chains (blue) comprising an N-terminal V_L_ and a single C_κ_ or C_λ_ domain. In addition, IgA1 and IgA2 can form dimers (dimeric SIgA1 depicted) by association with the J-chain (in green) which is stabilised by covalent binding of the secretory component (red). IgM can form pentamers in association with the J-chain as well as non-covalent interactions with secretory component (not depicted) to form SIgM.

**Figure 2:**
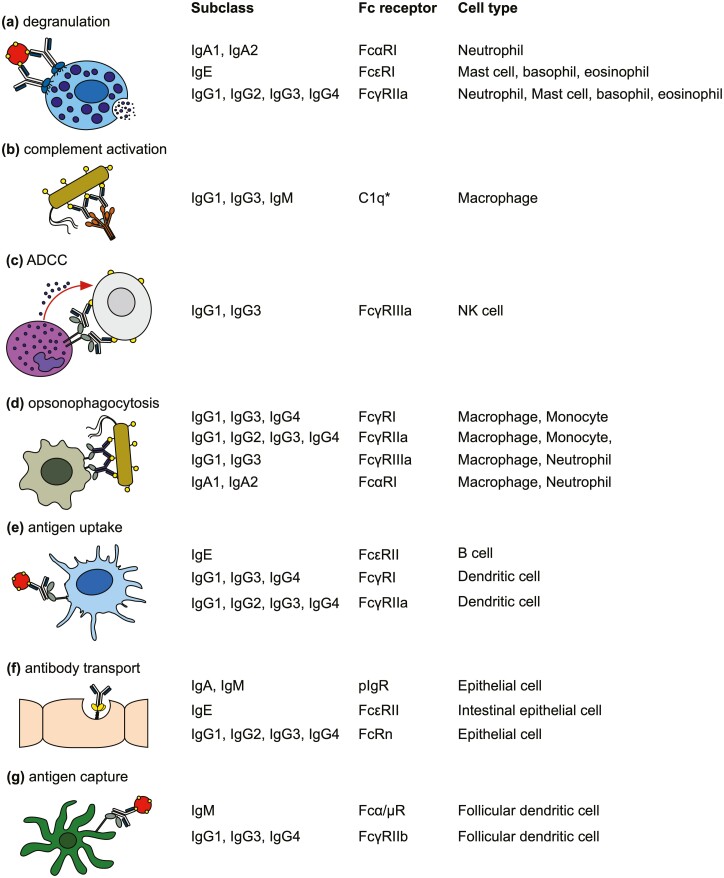
Fc-dependent antibody functions. Antibodies mediate a variety of effector functions via Fc-dependent interaction with receptors and proteins localised at different sites throughout the body. The most common functions are illustrated here, including **(a)** degranulation of innate myeloid cells, **(b)** complement-dependent activation resulting in direct pathogen lysis or enhanced phagocytosis, **(c)** antibody-dependent cellular cytotoxicity by NK cells, **(d)** opsonisation of pathogens to facilitate phagocytosis, **(e)** facilitated antigen uptake by antigen presenting cells, **(f)** receptor-mediated transport of antibody across mucosal barriers, and **(g)** capture of antigen for regulation of germinal centre responses. *C1q is not a receptor but can bind to antibody Fc and cross-link complement receptors on the surface of macrophages, thereby promoting complement-mediated phagocytosis.

All antibodies are made up of two identical heavy and light chains. The antigen binding fragment (Fab) interacts with the antigen, confers specificity, and is unique to each individual B cell clone. In humans, the crystallizable fragment (Fc) can be one of five isotypes (Fig. 1), each with distinct structural features that confer different effector functions. These functions are determined by interactions with receptors expressed on discrete populations of immune and non-immune cells that vary by lineage and across body tissues (reviewed in [4]). Classical Fc receptors (FcRs) can be broadly divided into activating or inhibitory depending on the presence of immunoreceptor tyrosine-based activation (ITAM) or inhibitory (ITIM) motifs in the cytoplasmic tail. Activating receptors including FcγRI, FcγRIIa, FcεRI, and FcαRI are typically expressed by myeloid cells and result in cellular stimulation, as well as promoting degranulation, cytotoxicity, and/or phagocytosis according to cell type (Fig. 2). Co-expression and cross-linking of inhibitory receptors can attenuate activating signals. In addition, non-classical FcRs such as C-type lectin receptors expressed by antigen-presenting cells (APCs) and various non-immune cells can mediate antigen capture, enhance APC activity, and mediate antibody transport (Fig. 2). In addition to FcRs, some antibody subclasses can bind the complement component C1q to initiate the complement cascade.

### IgD

IgD is the second least abundant antibody class in human serum after IgE and has a relatively short half-life. Despite being scarce in circulation, IgD plays an underappreciated role in mucosal immunity. IgD-producing plasma cells are derived from nasopharynx-associated lymphoid tissue and have restricted dissemination in the aerodigestive tract [5]. IgD derived from these cells displays extensive somatic hypermutation and recognizes important respiratory pathogens such as *Haemophilus influenzae* but also targets commensals and soluble dietary proteins [6, 7]. IgD does not interact with C1q or Fc receptors but instead binds to innate cells such as basophils and mast cells via galectin-9 and CD44. Receptor cross-linking triggers the release of IL-4 which promotes T follicular helper type 2-dependent responses, leading to the production of IgG and IgE [8]. Despite promoting IgE production, IgD also attenuates IgE-mediated mast cell and basophil degranulation [8], pointing to a role for IgD in maintaining homeostasis at mucosal barriers.

### IgM

IgM plays a significant role in initiating immune responses and pathogen clearance. This isotype is produced early after antigen encounter by short-lived extrafollicular plasma cells, but can also be generated by affinity-matured long-lived plasma cells [9]. The pentameric structure of IgM (Fig. 1) provides high avidity for antigen binding, which offsets the typically lower affinity of IgM relative to other subclasses. Antigen binding results in a conformational change in IgM structure, exposing a C1q-binding site that promotes highly efficient activation of complement. The unique structure of IgM also permits effective agglutination of intestinal microbiota which promotes microbe clearance and limits antigen penetration into the epithelium. In addition, natural IgM is produced spontaneously by distinct subsets of B cells in the absence of antigen stimulation or T cell help [10]. Natural IgM is germline encoded, usually polyreactive, and recognizes a broad range of antigens including self-molecules. This isotype also provides immediate early protection against infection, for example through effective virus neutralization [11]. In humans, a population of CD20^+^ CD27^+^ CD43^+^ CD70^−^ cells that are functionally equivalent to murine B1 cells produce natural antibodies. However, unlike mice the main source of human natural antibodies is marginal zone B cells. IgM comprises around 10% of serum antibodies but also has a key role in mucosal immunity, making up 10–20% of the plasma cell population in the human terminal ileum [12]. Polymeric IgM binds to pIgR via its J-chain to enable transport across the epithelium as secretory IgM. Secretory IgM, which is mostly derived from natural IgM, plays a role in maintaining tolerance of commensal bacteria (reviewed in [10]). The subject of IgM B cells is covered elsewhere in this issue [13]. Selective IgM deficiency is a rare disorder that can result in the recurrent upper respiratory tract and systemic infections, as well as less common allergic and autoimmune conditions [14]. IgM-containing immune complexes can bind to Fcα/μR, which in humans is primarily expressed on follicular dendritic cells [15]. Pentameric IgM associates with the protein apoptosis inhibitor of macrophages (AIM) to facilitate retention of immune complexes in germinal centres [16]. These complexes provide a key source of antigen for B cell maturation in germinal centre responses, thus highlighting an additional role for IgM in regulating adaptive immunity.

### IgA

Unlike most mammals, humans (and some primates) have two subclasses of IgA that differ in hinge structure (Fig. 1) and patterns of distribution around the body. IgA1 is present at a higher ratio in serum, while IgA2 is present at a higher ratio in the colon, and both are present in equal quantities at other mucosal surfaces. IgA is in fact the most abundant class of antibody with several grams produced each day, primarily in the gut. In humans, the majority of gut IgA+ plasma cells are derived from IgM memory B cells [17]. While most serum IgA is monomeric, mucosal IgA is dimeric and requires transport into the gut lumen by pIgR (the same mechanism as used by IgM), where it associates with the secretory component to stabilize the dimeric structure. Secretory IgA maintains intestinal homeostasis by binding to and coating diverse commensal microbes [18, 19], thus constraining them to the gut lumen. IgA does not bind to C1q so cannot activate complement. Instead, both dimeric and monomeric IgA bind to FcαRI (CD89) which is constitutively expressed on myeloid cells and mediates phagocytosis and ADCC [20, 21]. Secretory IgA does not bind FcαRI due to steric hindrance of the secretory component [22]. Cross-linking of FcαRI by IgA2-immune complexes results in signaling through the associated FcR γ subunit, leading to a pro-inflammatory response that drives cytokine production and NET formation by neutrophils. This response is not observed with IgA1-complexes, likely due to their different patterns of glycosylation, representing a key functional difference between IgA subclasses [23]. Although important in host defense, interactions between FcαR and IgA can also contribute to immune pathology in inflammatory diseases such as ulcerative colitis [24]. Conversely, the binding of monomeric IgA to FcαRI inhibits signaling through other FcRs [25] and acts as a negative regulator of inflammation. Selective IgA deficiency is one of the most common antibody deficiencies, affecting around 1 in 500 individuals. Although largely asymptomatic and thought to be partly compensated by sIgM [26, 27], ~20–30% of patients present with recurrent respiratory tract infections [28], mostly caused by encapsulated bacteria.

### IgG

As the most abundant class of antibody in serum, IgG plays a central role in systemic immunity and is the primary effector antibody raised in response to inflammation. The four IgG subclasses differ in hinge structure and CH2 domains (Fig. 1), resulting in divergent effector functions which are achieved via differential binding to Fcγ receptors and C1q [29]. Whereas IgG1 and IgG3 induce potent pro-inflammatory responses, IgG2 and IgG4 are more often associated with anti-inflammatory responses and/or tolerance. In addition to a central function in systemic immunity, IgG plays an important role at mucosal surfaces, particularly in the respiratory mucosa and genital tract, where concentrations of IgG exceed those of IgA [30]. All IgG subclasses are monomeric and therefore do not bind to pIgR. Instead, the IgG family of antibodies can bind to the neonatal Fc receptor (FcRn) which is expressed on epithelial cells and facilitates transport across mucosal barriers as well as the placenta. FcRn is also expressed on endothelial and myeloid cells where it functions to extend IgG half-life [31].

### IgG1

Approximately half of all serum antibodies are IgG1, which is a potent activator of complement and binds with high affinity to activating Fcγ receptors, thus making it a key player in humoral immunity. Given this abundance, specific IgG1 deficiency results in overall hypogammaglobulinemia and is associated with generalized susceptibility to infection [32]. IgG1 is the dominant subclass produced in response to many infectious diseases, but as a key mediator of inflammation, can also trigger host pathology. In patients with ulcerative colitis, a massive influx of IgG1-producing plasma cells into the gut mucosa leads to the formation of immune complexes that can trigger macrophage release of pro-inflammatory cytokines [33].

### IgG2

IgG2 makes up approximately 16% of total serum antibodies and almost all of the fraction that binds specifically to bacterial polysaccharides [34]. IgG2 does not bind to the high-affinity IgG receptor FcγRI and interacts only weakly with C1q, making it a poor activator of complement. The anti-bacterial effector function of IgG2 is instead mediated primarily through interactions with FcγRIIa on granulocytes, which promotes phagocytosis of encapsulated bacteria opsonized by IgG2 [35]. Accordingly, specific IgG2 deficiency (and polymorphisms in FcγRIIa) result in increased susceptibility to bacterial infections.

### IgG3

At just 4% of IgG in serum, IgG3 is far less abundant than IgG1 but appears very rapidly following viral infections, thus playing a key role in early immune defense (reviewed in [36]). IgG3 has the highest affinity for C1q [37] making it a potent activator of complement, as well as binding with high affinity to activating Fcγ receptors. IgG3 also has the longest hinge of all the subclasses, which provides a high degree of flexibility. The extended hinge of IgG3 potentiates the antiviral activity of the cytosolic antibody receptor TRIM21, which targets antibody-virus complexes for degradation and activates pro-inflammatory gene expression [38]. Accordingly, selective IgG3 deficiency is associated with recurrent upper respiratory tract infections, sinusitis, and pneumonia [39]. Conversely, increased serum IgG3 has been linked with disease activity in multiple sclerosis [40].

### IgG4

IgG4 antibodies are rarely induced in response to infections but instead arise in response to chronic antigen exposure, typically allergens and other non-infectious protein antigens. IgG4 is regarded as a non-inflammatory subclass since it does not bind to C1q and has a relatively low affinity for activating Fcγ receptors. The hinge of IgG4 has intra-chain bonds and displays weaker interactions between CH3 domains compared with other IgG subclasses [41]. This results in the random recombining of different IgG4 half molecules, thereby generating bi-specific antibodies that are functionally monovalent and thus unable to form immune complexes [42]. Despite clear associations with immune tolerance in the context of type 2 responses [43], IgG4 antibodies are a characteristic of a spectrum of fibrotic disorders termed IgG4-related disease (IgG4-RD). This pathology is associated with organ-specific infiltration of IgG4-producing plasmablasts [44], although it remains unclear whether IgG4 antibodies are actually involved in the IgG4-RD disease process. Albeit less marked, tissue infiltration of IgG4-producing plasmablasts has also been reported in eosinophilic oesophagitis (EoE), with approximately 76% of patients displaying extracellular IgG4 deposits [45], and biopsies containing increased levels of IgG4 reactive to common EoE trigger foods [46].

### IgE

Despite being the least abundant antibody class in human serum and displaying the shortest half-life, IgE has a significant and highly prevalent impact on human health as the central mediator of allergic disease. The potent effector functions of monomeric IgE are a consequence of high affinity for FcεRI expressed on granulocytes, which increases its half-life to several weeks [47]. IgE is produced primarily in mucosal tissue where it is sequestered on FcεRI-expressing cells [48]. Allergen cross-linking of FcεRI-IgE on the surface of granulocytes results in rapid activation and release of various mediators including histamine, tryptase, and pro-inflammatory cytokines. Apart from its deleterious effects in allergic disease, IgE is also associated with type 2 immunity and fulfils protective functions at barrier sites. Classically, IgE responses are associated with helminth infection [49, 50], but more recently IgE has been shown to also confer protection against venoms, toxins, and even tumour formation [51–53]. Consistent with this concept, IgE deficiency has previously been linked with increased rates of malignancy [54, 55], pointing to complex biology that is not yet fully understood. Indeed, the role of self-reactive IgE in autoimmune diseases such as chronic urticaria, systemic lupus erythematous, and bullous pemphigoid [56–59], has gained increasing attention to become an active area of research. IgE complexes can also bind to FcεRII (CD23) on APCs to enhance antigen-presenting capacity by several orders of magnitude [60], leading to marked amplification of immune responses.

## Regulation of antibody production

**Figure 3:**
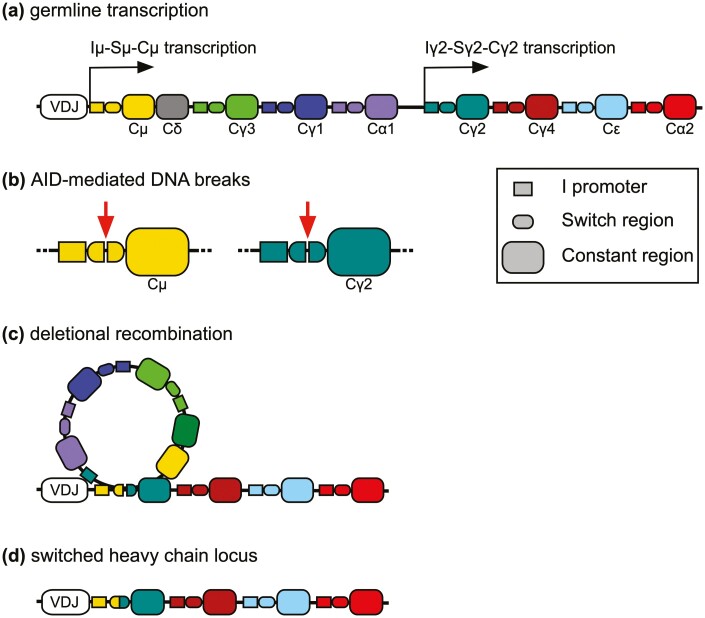
Schematic overview of class switch recombination (CSR). **(a)** Signals from T cell-derived cytokines and CD40 enhance germline transcription from the donor (Iμ depicted) and downstream acceptor (Iγ2 depicted) I_H_ promoters. **(b)** The same signals induce expression of AID which can bind to single-stranded DNA at transcriptionally active switch regions to mediate the formation of double-stranded breaks. **(c)** Repair of double-stranded breaks results in recombination and deletion of the intervening DNA **(d)**, allowing CSR to replace the constant region downstream of the VDJ exon and thus permitting transcription of a new antibody subclass.

Class switching is a tightly regulated, irreversible process that exchanges the constant region gene by deletional recombination (Fig. 3). It remains unclear which factors dictate whether a given B-cell will switch or not. CSR is a rare event initiated by germline transcription of the target immunoglobulin genes from the intervening (I) exon under the direction of a promoter. Switch (S) regions upstream of each constant region gene (except Cδ) contain tandem repeat sequences that are targeted by activation-induced cytidine deaminase (AID). This enzyme is induced by the combination of IL-4 and CD40 signaling, leading to the deamination of cytosines in donor and acceptor S regions to generate uracils. This triggers a mismatch repair mechanism that introduces single-stranded DNA breaks that are subsequently converted into double-stranded breaks (Fig. 3), leading to non-homologous end repair. Despite the absence of a switch region in Cδ, a subset of tonsil B cells can undergo class switching to IgD by virtue of a short intronic region between Cμ and Cδ which acts as an acceptor site for IgM to IgD switching [61]. Active transcription is essential for class switching to take place since this allows AID to bind single-stranded DNA at the relevant target sequences within switch regions. The I_H_ promoters have cytokine-responsive elements that differ between subclasses, hence the efficiency of CSR directly correlates with germline transcription [62]. For example, single nucleotide differences in the Iγ1 and Iγ4 promoters confer divergent responses to CD40 signaling, resulting in much lower germline transcription from Iγ4 [63]. In addition, while all switch regions have transcription factor binding sites responsive to IL-4 and CD40 signaling, these vary in length and in the number of tandem repeats between individual subclasses. The likelihood of a switch event occurring therefore differs according to the length and composition of I and S region sequences [63–66]. Unlike Iγ1-4 and Iε, Iα1, and Iα2 exhibit binding elements that are responsive to TGFβ1-inducible transcription factors, namely SMAD proteins and RUNX3 [67]. Consequently, IgA class switching can be induced by TGFβ1 in addition to IL-4. Although class switching is typically associated with T-dependent responses, CSR can also be induced by TNF superfamily members BAFF and APRIL binding to TACI, without the need for CD40 or T-cell help [68]. Class switching has also been observed in tertiary lymphoid structures, which form transiently at sites of inflammation and support clonal expansion and B cell maturation (reviewed in [69])

## Impact of class switching on the germinal centre response and cell fate

Following activation and interaction with cognate T cells, some B cells expand within extrafollicular foci to give rise to short-lived plasma cells and extrafollicular memory B cells, whereas others form germinal centres. The germinal centre response involves clonal expansion and affinity-based selection of antigen-specific B cells, followed by their differentiation into either long-lived memory B cells or plasma cells. The fate of germinal centre B cells is intricately linked to the B cell receptor, and affinity for antigen plays a key role in B cell fate selection. Higher affinity binding has been linked to preferential differentiation towards plasma cells, either due to cell-intrinsic mechanisms linked with BCR signaling or because of improved antigen capture and increased competitiveness for T cell help (since prolonged contact with T follicular helper cells increases B cell expression of key transcription factors necessary for plasma cell differentiation; reviewed in [70]).

Although germinal centre B cells have higher thresholds for activation than naïve B cells [71], those that express switched isotypes have lower thresholds for activation compared to unswitched B cells [72]. In addition, class switched germinal centre B cells display gene expression patterns linked with germinal centre homing and retention that favour ongoing affinity maturation and T-cell help [73]. Since antibody class switching usually occurs early after antigen encounter and prior to the formation of the GC response [73–76], BCR isotype can influence the fate of germinal centre B cells. The membrane immunoglobulins (mIg) of both IgM and IgD have a short cytoplasmic domain only 3 amino acids long, whereas mIgG1-4 has a conserved cytoplasmic tail of 29 amino acids, the mIgE tail comprises 28 amino acids, and the tails of mIgA1 and mIgA2 include 14 amino acids [77]. Ig-α (CD79a) and Ig-β (CD79b) are the signaling elements for the BCR. The intracellular domains of the Igα-Ig-β heterodimer contain an ITAM which recruits Syk to initiate a signaling cascade in response to receptor cross-linking. Although all mIg signal through Ig-α-Ig-β, mIgG and mIgE have a conserved immunoglobulin tail tyrosine (ITT) which permits inducible phosphorylation by ITAM-bound Syk, thereby reducing the threshold for activation four-fold and augmenting plasma cell differentiation. Interestingly, while mIgA lacks an ITT motif, selective depletion of mIgA results in a near total absence of IgA-secreting PC [78].

The short-lived plasma cells generated in extrafollicular responses secrete antibodies that help combat acute infections, but long-lived plasma cells (LLPC) derived from germinal centres are the primary source of high-affinity, class-switched serum antibodies that underpins protective immunity. Human LLPC can survive for decades and continually secrete high concentrations of antibody [79]. Plasma cell longevity is dependent on migration to survival niches which include the bone marrow and secondary lymphoid tissues. Of note, while surface BCR is widely considered to be downregulated upon plasma cell differentiation, those expressing IgM, IgA and IgE still display a functional BCR [80–82], the significance of which remains an active area of investigation. Depending on the context in which differentiation occurs, the location and to some extent phenotype of LLPC are associated with isotype [77]. Homing of IgA+ plasma cells to mucosal tissues is controlled by a combination of integrins such as α4β7 and chemokine receptors, with CCR10 directing migration to the bronchial, salivary, oral, and intestinal epithelium, whereas CCR9 directs migration to the small intestine [83]. Recruitment of IgG+ plasma cells to inflamed tissues is instead mediated via CXCR3, which serves as a useful marker of detrimental responses in disorders such as rheumatoid arthritis [84].

## Role of subclass in long-lived humoral memory

Along with plasma cells, memory B cells are an essential component of long-lived protective immunity, generating rapid responses during secondary antigen exposure. Memory B cells express a BCR at the cell surface and display extensive heterogeneity which has been linked to isotype. This is most evident when comparing switched and unswitched memory B cells. Although unswitched cells comprise a significant portion of extrafollicular-derived cells, transcriptional profiling of switched versus unswitched populations has revealed marked functional diversity despite equivalent levels of somatic hypermutation (a marker of GC experience) [73]. Crucially, unswitched memory B cells are more likely to reinitiate GC responses following secondary activation [85–87], and in keeping with this role, they also express genes associated with antigen presentation and cytokine signaling [73]. Conversely, switched memory B cells are more likely to undergo rapid differentiation into plasma cells following secondary activation [73, 85–87].

Different subsets of memory B-cell display functional heterogeneity that influences their ability to expand, migrate, and differentiate the following activation. These include variable expression of surface receptors and secreted factors that modulate responses to antigens, influence their localisation, and alter interactions with other cell types in the local environment. For example, increased expression of CXCR5 on IgG3+ memory B cells was reported in patients with multiple sclerosis [40]. In contrast, IgG4+ B cells display reduced levels of CXCR4 and CXCR5 and do not express CCR7, which restricts their entry into secondary lymphoid organs [88–90]. IgG4+ memory B cells also display relatively lower expression of complement receptor 2 (CR2/CD21). CR2 enhances signaling through the BCR [91], thereby lowering the threshold for B cell activation and promoting survival, indicating that IgG4+ B cells have different responsiveness to antigens as well as distinct patterns of migration. While current evidence for more refined phenotypic specialization based on antibody subclass is limited, the few studies conducted on memory B cell populations in disease states strongly support this concept, hence further work in this area is highly warranted.

Most analyses of memory B cell populations focus on circulating populations, but there is increasing appreciation for the role of tissue-resident memory B cells in immune surveillance (reviewed in [92]). As highlighted above, the localization of antibody responses is crucial for effective immunity. Tissue-resident memory B cells are non-circulating, long-lived, and can be defined by high levels of CD69 [93, 94]. They reside in tissues in a non-motile, quiescent state, but upon secondary infection they rapidly accumulate and differentiate into local plasma cells that provide high concentrations of protective antibody. The microenvironment in which tissue-resident B cells reside can greatly influence phenotype and functional responses via modulation of antibody subclass. For example, in autoimmune blistering disease the balance between IgG1 and IgG4 expression determines the pathologic potential of the autoantibodies produced [95]. It is therefore crucial to understand how the functional properties of tissue-resident B cells differ from those present in lymphoid tissue and peripheral blood in health and disease states, as well as elucidate the role played by the tissue microenvironment in shaping local antibody responses.

Memory B cells retain the ability to class switch, which has major implications for secondary responses. Antibody repertoire analysis of peripheral blood B cells has revealed that while the majority of switch events occur from IgM to IgG1 and IgA1, distinct hierarchies of class switching exist that cannot be explained by the relative abundance of individual subclasses [96]. Switching to downstream subclasses (IgG2, IgG4, IgE, and IgA2) rarely occurs through direct switching from IgM; for example, most IgA2 is derived through sequential switching from IgA1 or IgG2. Whether this reflects phenotypic differences in memory B cell responsiveness or common pathways of subclass-specific switching remains to be determined. The difference in BCR responsiveness between switched and unswitched memory B cells can be explained by cell-intrinsic differences, for example, the composition of the cytoplasmic tail as described above. However differential expression of surface receptors by subpopulations of memory B cells could indicate additional specialisation based on isotype. Whether this relates to the conditions under which initial antigen priming occurred warrants further investigation, since this could have important implications for future vaccine design.

## Summary and future perspectives

Collectively, the different antibody subclasses provide protective immunity and maintain homeostasis. While there are clear overlaps in effector function, each isotype performs specialist roles in terms of timing, distribution, and specificity of a response, as highlighted by the clinical phenotypes of selective antibody deficiency. The regulation of subclass production is therefore critical for the generation of an appropriate antibody response to specific pathogens. The contribution of antibodies to disease also continues to gain attention with the discovery of new roles in conditions not previously thought to be immune-mediated, such as fibromyalgia [97] and cardiovascular disease [98]. Similarly, trials of B cell-targeted therapies have drawn attention to previously overlooked roles in multiple sclerosis [99, 100]. Advances in single-cell technologies now allow us to examine the phenotypic and functional properties of B cells in parallel with antibody repertoires at scale, which is revealing a diversity of populations that was not previously recognized. As these technologies become more accessible, it will be possible to address many unresolved questions about how antibody responses are regulated at a cellular level. A key issue relates to the regulation of antibody responses in peripheral tissues and how the microenvironment in inflamed versus non-inflamed tissues influences B cell function. Similarly, fundamental questions about antibody subclass-based phenotypic specialization can now be addressed. Understanding how antibody class affects memory B cell capacity to undergo class switching and selection is essential to understanding secondary immunity and therefore has major implications for the clinical manipulation of B cell responses to benefit human health.

## Data Availability

N/A

## Abbreviations

ADCC: antibody dependent cellular cytotoxicity
AID: activation induced cytidine deaminase
AIM: apoptosis inhibitor of macrophages
APC: antigen presenting cell
APRIL: a proliferation-inducing ligand
BAFF: B-cell activating factor
BCR: B cell receptor
CSR: class switch recombination
EoE: eosinophilic eosophagitis
Fab: fragment antigen binding
Fc: fragment crystallisable
FcR: Fc receptor
GC: germinal centre
Ig: immunoglobulin
ITAM: immunoreceptor tyrosine-based activation motif
ITIM: immunoreceptor tyrosine-based inhibitory motif
LLPC: long-live plasma cell
NET: neutrophil extracellular trap
TACI: transmembrane activator and CAML interactor
TRIM21: tripartite motif containing-21
